# An In Vivo Inflammatory Loop Potentiates KRAS Blockade

**DOI:** 10.3390/biomedicines10030592

**Published:** 2022-03-03

**Authors:** Kristina A. M. Arendt, Giannoula Ntaliarda, Vasileios Armenis, Danai Kati, Christin Henning, Georgia A. Giotopoulou, Mario A. A. Pepe, Laura V. Klotz, Anne-Sophie Lamort, Rudolf A. Hatz, Sebastian Kobold, Andrea C. Schamberger, Georgios T. Stathopoulos

**Affiliations:** 1 Comprehensive Pneumology Center (CPC), Institute of Lung Health and Immunity (LHI), Helmholtz Zentrum München, 81377 Munich, Germany; arendtka@outlook.com (K.A.M.A.); christin.henning@tum.de (C.H.); giotopoulou.g@gmail.com (G.A.G.); mario.pepe1985@libero.it (M.A.A.P.); laura.klotz@med.uni-heidelberg.de (L.V.K.); as.lamort@gmail.com (A.-S.L.); 2 Member of the German Center for Lung Research (DZL), 35392 Gießen, Germany; 3 Laboratory for Molecular Respiratory Carcinogenesis, Department of Physiology, Faculty of Medicine, University of Patras, 26504 Rio, Greece; ntaliarda@upatras.gr (G.N.); vasilisarmenis91@gmail.com (V.A.); danai.kati@gmail.com (D.K.); 4 Center for Thoracic Surgery, University Hospital, Ludwig-Maximilians-Universität (LMU), 81377 Munich, Germany; r.hatz@asklepios.com; 5 Asklepios Medical Center, 82131 Gauting, Germany; 6 Center of Integrated Protein Science Munich (CIPS-M), Division of Clinical Pharmacology, Department of Medicine IV, Klinikum der Universität München, Lindwurmstraße 2a, 80337 Munich, Germany; sebastian.kobold@med.uni-muenchen.de

**Keywords:** deltarasin, IL-1β, IL1R1, KRAS, KRAS mutation, KRAS^G12C^, inflammation, lung cancer

## Abstract

KRAS (KRAS proto-oncogene, GTPase) inhibitors perform less well than other targeted drugs in vitro and fail clinical trials. To investigate a possible reason for this, we treated human and murine tumor cells with KRAS inhibitors deltarasin (targeting phosphodiesterase-δ), cysmethynil (targeting isoprenylcysteine carboxylmethyltransferase), and AA12 (targeting KRAS^G12C^), and silenced/overexpressed mutant KRAS using custom-designed vectors. We showed that *KRAS*-mutant tumor cells exclusively respond to KRAS blockade in vivo, because the oncogene co-opts host myeloid cells via a C-C-motif chemokine ligand 2 (CCL2)/interleukin-1 beta (IL-1β)-mediated signaling loop for sustained tumorigenicity. Indeed, *KRAS*-mutant tumors did not respond to deltarasin in C-C motif chemokine receptor 2 *(Ccr2)* and *Il1b* gene-deficient mice, but were deltarasin-sensitive in wild-type and *Ccr2*-deficient mice adoptively transplanted with wild-type murine bone marrow. A KRAS-dependent pro-inflammatory transcriptome was prominent in human cancers with high *KRAS* mutation prevalence and poor predicted survival. Our findings support that in vitro cellular systems are suboptimal for anti-KRAS drug screens, as these drugs function to suppress interleukin-1 receptor 1 (IL1R1) expression and myeloid IL-1β-delivered pro-growth effects in vivo. Moreover, the findings support that IL-1β blockade might be suitable for therapy for *KRAS*-mutant cancers.

## 1. Introduction

Since its discovery, the Kirsten rat sarcoma virus (KRAS) proto-oncogene GTPase (encoded by the human *KRAS* and the murine *Kras* genes) has become the holy grail of anticancer therapy [1,2]. The KRAS oncoprotein possesses a unique molecular structure that potentiates it as a driver of multiple cancer cell hallmarks (including proliferation, migration, metastasis, angiogenesis, inflammation, and apoptosis evasion), but also renders it non-actionable due to the absence of a druggable deep pocket [2,3]. KRAS point mutations that constitutively activate GTPase function occur most frequently in codons 12, 13, and 61, and are particularly frequent in pancreatic (70%), colorectal (35%), and lung (20%) adenocarcinomas [3,4]. However, full KRAS GTPase activity and downstream signaling additionally prerequires its integration into the cell membrane, which is facilitated by the post-translational lipidation and membrane transport of KRAS by various enzymes, such as farnesyltransferase (FT), geranylgeranytransferase (GGT), isoprenylcysteine carboxyl methyltransferase (ICMT), phosphodiesterase-δ (PDEδ), and others [3,5]. To this end, therapeutic attempts to inhibit KRAS lipidation by targeting FT/GGT/ICMT were recently coupled with the development of PDEδ blockers and of allosteric and covalent inhibitors of mutated KRAS^G12C^ [6,7,8,9].

Despite coordinated efforts [1], anti-KRAS drug discovery lags behind other oncogene targets [3], and only one single KRAS^G12C^ inhibitor (sotorasib) was recently approved by the FDA for non-small-cell lung cancer (NSCLC) [10,11]. In addition to molecular structural considerations [5], the mode of KRAS oncogenic functions could be a reason for this. To this end, Janes and collaborators recently reported a discordance between the in vitro and the in vivo effects of a newly developed covalent KRAS^G12C^ inhibitor [9]. This observation is relevant to other reports describing how KRAS-dependence is linked to signatures of intravital-restricted processes such as inflammation and epithelial-to-mesenchymal transition [12,13,14] and how pro-inflammatory properties of *KRAS* mutations potentiate malignant pleural effusions in mice [15,16].

Here, we hypothesized that KRAS effects and druggability are preferentially at play in vivo. We tested the efficacy of three different KRAS inhibitors with divergent modes of action in vitro and in vivo using a battery of 30 natural and transduced human and murine cancer cell lines and four different methods to integrally assess tumor cell growth. We consistently show that KRAS inhibitors exerted ubiquitous in vitro effects irrespective of cellular *KRAS* mutation status, but were specifically effective against *KRAS*-mutant tumors in vivo. Using transcriptome analyses of cell lines expressing endogenous or exogenous wild-type or mutant *Kras* alleles, *Ccr2* (C-C motif chemokine receptor 2) and *Il1b* (interleukin-1 beta, IL-1β) gene-deficient mice, as well as adoptive bone marrow transfer, we show that mutant KRAS established a proinflammatory CCL2 (C-C motif chemokine ligand 2)/IL-1β-mediated signaling loop to host myeloid cells in vivo, which is required for *KRAS*-mediated tumorigenicity and, importantly, for specific KRAS inhibitor efficacy. The *KRAS/CCL2/IL1B* transcript signature was further shown to be enriched in human tumors with higher *KRAS* mutation frequencies and to portend poor survival. Our data show that intact inflammatory tumor-to-host interactions were required for full KRAS inhibitor efficacy and imply that in vitro drug screens might not be optimal for KRAS inhibitor discovery.

## 2. Materials and Methods

### 2.1. Cell Culture 

NCI-H358, NCI-H358M, NCI-H460, NCI-H520, NCI-H1299, NCI-H1944, NCI-H3122 (referred to hereafter omitting NCI), EKVX, A549, LLC, B16F10, and PANO2 cell lines were obtained from the National Cancer Institute (Frederick, MD, USA); MC38 cells were a gift from Dr. Timothy S. Blackwell (Vanderbilt University; Nashville, TN, USA) and AE17 cells from Dr. YC Gary Lee (University of Western Australia, Perth, Australia) [15,16,17]. FULA1 (*FVB* urethane-induced lung adenocarcinoma 1) and CULA (*C57BL/6* urethane-induced lung adenocarcinoma) cell lines were isolated from the lungs of *FVB* and *C57BL/6* mice, respectively, harboring primary lung adenocarcinomas induced by urethane [15,18,19]. Human and murine cell lines were cultured in Roswell Park Memorial Institute (RPMI)-1640 and Dulbecco’s Modified Eagle Medium (DMEM), respectively, both supplemented with 10% FBS and 100 IU/mL penicillin/streptomycin, and were maintained in a humidified incubator at 37 °C with 95% air/ 5% CO_2_. Cell lines were authenticated annually using the short tandem repeat method and were tested negative for *Mycoplasma* spp. biannually with the MycoAlert Mycoplasma Detection Kit (LONZA; Verviers, Belgium).

### 2.2. Drugs 

Deltarasin (CAS #1440898-82-7; Tocris Bio-Techne #5424; Wiesbaden-Nordenstadt, Germany), KRAS^G12C^ inhibitor 12 (AA12; CAS #1469337-95-8; Selleckchem #S7331; Houston, TX, USA), and cysmethynil (CAS #851636-83-4; Cayman Chemicals #14745; Ann Arbor, MI, USA) were dissolved in dimethylsulfoxide (DMSO) until 10 mM stock concentration and stored at −80 °C. For in vitro and in vivo experiments, drugs were further diluted in saline. Equimolar DMSO solutions were used as controls. 

### 2.3. Cellular Assays 

In vitro cell proliferation was determined using the water soluble tetrazolium-1 [2-(4-iodophenyl)-3-(4-nitrophenyl)-5-(2,4-disulphophenyl)-2H-teterazolium; WST-8] assay (Bimake; Munich, Germany). For this, 3000 cells/well were plated in triplicates in 96-well plates in 5% FBS-containing media and allowed to adhere overnight, followed by treatment with different drug concentrations. WST-8 reagent was added 72 h later according to the manufacturer’s protocol and absorbance at 450 nm was measured 1–4 h later on a TECAN Sunrise microplate reader (Männedorf, Switzerland). For the colony formation assay, 300 cells were plated in triplicates in 6-well plates in 5% FBS-containing media and were treated 24 h later with 1–2 µM deltarasin; media were replaced with drug-free media 72 h later, and cells were incubated until ≤ 50 colonies formed. Colonies were fixed with 80% ethanol, stained with 0.5% crystal violet, counted and photographed. All cellular experiments were independently repeated at least twice.

### 2.4. Western Immunoblotting

Cellular protein lysates were prepared using Radio-Immunoprecipitation Assay (RIPA) buffer containing phosphatase/protease inhibitor cocktail (Thermo Fisher; Waltham, MA, USA), separated via SDS-PAGE, and transferred to nitrocellulose membranes according to standard protocols. Anti-total (t)-extracellular-signal regulated kinase (ERK), anti-phospho (p)-ERK, and anti-glyceraldehyde 3-phosphate dehydrogenase (GAPDH) antibodies were obtained from Santa Cruz Biotechnology (Houston, TX, USA) (Supplementary Table S1).

### 2.5. Constructs

Short-hairpin (sh) RNA-mediated *Kras*-silenced (sh*Kras*) LLC, AE17, and MC38 cells, as well as B16F10 and PANO2 cells overexpressing custom-made plasmid encoding *Kras*^G12C^ (p*Kras*^G12C^; Addgene #64372; GFP-KrasG12C_2B_puro), were produced as described elsewhere [15]. H3122 and EKVX cells were stably transfected with p*Kras*^G12C^ or its homologous GFP backbone plasmid without *Kras*^G12C^ (p*C*; Addgene #64336; Bicistronic_GFP_ires_puro) using previously established methods (Supplementary Figure S5) [15]. All plasmids were produced in-house, deposited, validated, and re-purchased from Addgene (Watertown, MA, USA). Lentiviral shRNA targeting murine C-C motif chemokine ligand 2 *(Ccl2)* was purchased from Santa Cruz Biotechnology (random control shRNA sc-108080-V, GFP control shRNA sc-108084-V, murine sh*Ccl2* sc-43914-SH). For stable shRNA and plasmid transfection, 10^5^ tumor cells in 6-well culture vessels were transfected with 5 μg DNA using XFect (Takara; Kusatsu, Japan) and clones were selected with puromycin (2–10 μg/mL).

### 2.6. Mice

*FVB/NJ* (*FVB*; #001800), *C57BL/6J* (*C57BL/6*; #000664), B6.129P2-*Cxcr1*^tm1Dgen/J^ (*Cxcr1^−/−^*, C-X-C motif chemokine receptor 1; #005820) [17], B6.129S4-*Ccr2*^tm1Ifc/J^ (*Ccr2*^−/−^, C-C motif chemokine receptor 2; #004999) [16], B6.129S2(C)-*Cxcr2*^tm1Mwm/J^ (*Cxcr2*^+/−^, C-X-C motif chemokine receptor 2; #006848) [17], and B6(Cg)-*Rag2*^tm1.1Cgn^/J (*Rag2*^−/−^, recombination activating gene 2; #008449) mice were obtained from Jackson Laboratory (Bar Harbor, ME, USA) and *Il1b*^tm1Yiw^ mice (*Il1b*^−/−^, interleukin-1 beta; MGI #2157396) [20] were a kind gift from Dr. Yoichiro Iwakura (Tokyo University of Sciences; Tokyo, Japan). All mice were bred at the Center for Animal Models of Disease of the University of Patras (Patras, Greece). *Ccr2*^−/−^ mice were back-crossed to the *FVB* strain for > F12. Experimental mice were weight- (20–30 g), sex-, and age- (6–12 weeks) matched; both female and male mice were used and 284 mice were enrolled in total. In more detail, 25 *FVB* (21 for tumor experiments and 4 as bone marrow donors), 151 *C57BL/6* (all for tumor experiments), 15 *Cxcr1*^−/−^ (all on the *C57BL/6* background for tumor experiments), 34 *Ccr2*^−/−^ (12 on the *C57BL/6* and 18 on the *FVB* backgrounds for tumor experiments and 4 on the *FVB* background as bone marrow donors), 12 *Cxcr2*^+/−^ (all on the *C57BL/6* background for tumor experiments), 32 *Rag2*^−/−^ (all on the *C57BL/6* background for tumor experiments), and 15 *Il1b*^−/−^ (all on the *C57BL/6* background for tumor experiments) mice were used.

### 2.7. In Vivo Tumor Models and Drug Treatments

For in vivo injections, 10^6^ cells suspended in 50 µL PBS were implanted subcutaneously (sc) in the rear flank. Tumor dimensions (length, L; width, W; depth, D) were monitored serially using calipers and tumor volume (V) was calculated as V=π∗L∗W∗D/6. Drug treatments were initiated when tumors reached 100 mm^3^ volume and consisted of daily intraperitoneal (ip) deltarasin (15 mg/Kg in 100 μL saline 1% DMSO) or 100 μL saline 1% DMSO. Animals were monitored daily for sickness and were euthanized using CO_2_ when in distress or when tumors reached 2–3 cm^3^ volume, whichever came first.

### 2.8. Microarrays, PCR, GSEA, and Kaplan-Meier Analyses

Isogenic cell line doublets stably expressing sh*C* or sh*Kras* (LLC, MC38, and AE17 cells) and p*C* or p*Kras*^G12C^ (PANO2 and B16F10 cells) were generated as described elsewhere [15]. Benign samples including whole murine lungs, tracheal epithelial cells (TEC; cultured out from murine tracheas), and bone marrow-derived macrophages (BMDM; cultured from murine bone marrow via weekly incubation with 20 ng/mL M-CSF (macrophage colony-stimulating factor)) and mast cells (BMMC; cultured from murine bone marrow via monthly incubation with 100 ng/mL IL-3 plus KITL (kit ligand) were prepared as described elsewhere [16,19,20]. Cellular RNA was isolated using Trizol (Thermo Fisher), followed by RNAeasy column purification and genomic DNA removal (Qiagen; Hilden, Germany). For each analysis, 1 μg RNA was reverse-transcribed using oligo(dT)_18_ and the iScript Advanced cDNA synthesis kit for RT-qPCR (Bio-Rad Laboratories; Hercules, CA, USA). *Il1r1/IL1R1* (interleukin-1 receptor 1) and *Gapdh/GAPDH* qPCR was performed using specific primers (Supplementary Table S2) and Lightcycler 480 Sybr Green I Master Mix (Roche Diagnostics; Mannheim, Germany) in a Lightcycler 480 II (Roche Diagnostics). Ct values from triplicate reactions were analyzed using the 2^−ΔCT^ method as detailed elsewhere [17]. mRNA abundance was determined relative to *Gapdh/GAPDH* and is given as 2^−ΔCT^ = 2^−(Ct of *Il1r1/IL1R1*)−(Ct of *Gapdh/GAPDH*)^. Mouse microarrays were obtained as described elsewhere [15,16,17]. Briefly, triplicate cultures of 10^6^ cells were subjected to RNA extraction as above, 5 μg of pooled total RNA were tested for RNA quality on an ABI2000 Bioanalyzer (Agilent; Santa Clara, CA, USA), labelled, and hybridized to GeneChip Mouse Gene 2.0 ST arrays (Affymetrix; Santa Clara, CA, USA). Analyses using Affymetrix Expression/Transcriptome Analysis Consoles consisted of normalization of all arrays together using a Lowess multi-array algorithm, intensity-dependent estimation of noise for statistical analysis of differential expression, and unsupervised hierarchical clustering of microarray data and WikiPathway analysis. Murine microarray data are publicly available at the Gene Expression Omnibus (GEO) database (http://www.ncbi.nlm.nih.gov/geo/; Accession ID: GSE58190; last accessed: 4 March 2019). Gene set enrichment analyses (GSEA) were performed using publicly available Human Gene 1.0 ST microarray data obtained from GEO. The following datasets were used: GSE31852, with gene expression profiles of 121 biopsies from patients with lung adenocarcinoma (LUAD) with *EGFR* (epidermal growth factor receptor) (*n* = 17), *KRAS* (*n* = 21), or neither of the two (*n* = 83) mutations (Biomarker-integrated Approaches of Targeted Therapy for Lung Cancer Elimination (BATTLE) trial); GSE43458, with gene expression profiles of LUAD from smokers and never-smokers (*n* = 40 each), as well as normal lung tissue from never-smokers (*n* = 30), also from the BATTLE trial; and GSE103512, with gene expression profiles of breast (*n* = 65), colorectal (*n* = 55), and non-small-cell lung (*n* = 60) cancer patients from a Roche dataset. Kaplan-Meier analyses were performed using KM-plotter (http://www.kmplot.com; last accessed: 15 October 2021) [21]. All patients were included and overall survival and all stages/grades were set as parameters.

### 2.9. ELISA

Murine and human CCL2 levels of cell culture supernatants were detected using appropriate ELISA kits (Peprotech; London, UK). For sample preparation, cells were incubated with IC_60_ deltarasin for 72 h before collection of cell-free supernatants for CCL2 measurements and whole cellular lysates for normalization of CCL2 levels to total cellular protein.

### 2.10. Immunofluorescence

Paraffin-embedded mouse tissue blocks were cut into 3 µm-thick sections, deparaffinized via ethanol gradient, rehydrated, and boiled in sodium citrate pH 6.0 for 10 min for antigen retrieval. After post-fixation and permeabilization, tissue sections were co-stained with either AlexaFluor488-conjugated mouse monoclonal anti-IL-1β antibody and rabbit polyclonal anti-CCR2 antibody or AlexaFluor488-conjugated mouse monoclonal anti-IL1R1 antibody and rabbit polyclonal anti-CCL2 antibody (Supplementary Table S2). After counterstaining with 300 nM 4′,6-diamidino-2-phenylindole (DAPI), slides were evaluated on an AxioImager.M2 (Zeiss; Jena, Germany) and digital images were processed with Fiji academic software (https://fiji.sc/; last accessed: 15 October 2021). Control stains were carried out with isotype controls for normal mouse IgG1/ IgG2a (Alexa Fluor^®^ 488 conjugated; sc-3891/sc-3890) and secondary antibody only.

### 2.11. Bone marrow replacement

For adoptive bone marrow transplants (BMT), bone marrow cells were flushed from both femurs and tibias of wild-type (*WT*) or *Ccr2*^−/−^ mice (all back-crossed >F12 to the *FVB* background) using fully supplemented DMEM. *Ccr2*^−/−^ mice (all *FVB*) received 1 × 10^7^ bone marrow cells intravenously (iv) from *WT* or *Ccr2*^−/−^ mice 12 h after total-body irradiation (900 Rad), as described elsewhere [16,17,20]. One mouse in each experiment was not engrafted and was observed until moribund on days 5–15 post-irradiation. One month was allowed for full bone marrow reconstitution of chimeras prior to tumor cell injections.

### 2.12. Statistics

Sample size was calculated using G*power (http://www.gpower.hhu.de/; last accessed: 15 October 2021) [22]. In specific, we set out to determine the biologically (>50%) and statistically (α = 0.05; β = 0.20) significant differences between two unmatched independent groups with SD ~30% of mean using two-tailed *t*-tests, yielding *n* = 7/group. Hence, experiments with *n* = 5 mice/group were considered in batches until the achievement of probability (*p*) < 0.05 with α < 0.05 or *p* > 0.05 with β < 0.20, whichever came first. Two-way ANOVA was employed to achieve further reduction. Results are given as mean ± SD. Sample size (*n*) refers to biological replicates. Differences between means were assessed using one-way or two-way ANOVAs with Bonferroni post-tests. Fifty- and sixty-percent inhibitory concentrations (IC_50/60_) were calculated using nonlinear regression, a logarithmic inhibitor-response model, unweighted least squares regression without outlier elimination and constraints, and extra sum-of-squares F-test comparisons. *p* < 0.05 was considered significant. Statistics and plots were calculated on Prism versions 5.0, 6.0, and 8.0 (GraphPad; San Diego, CA, USA).

## 3. Results

### 3.1. Mutation-Independent Effects of KRAS Inhibitors In Vitro

**Figure 1:**
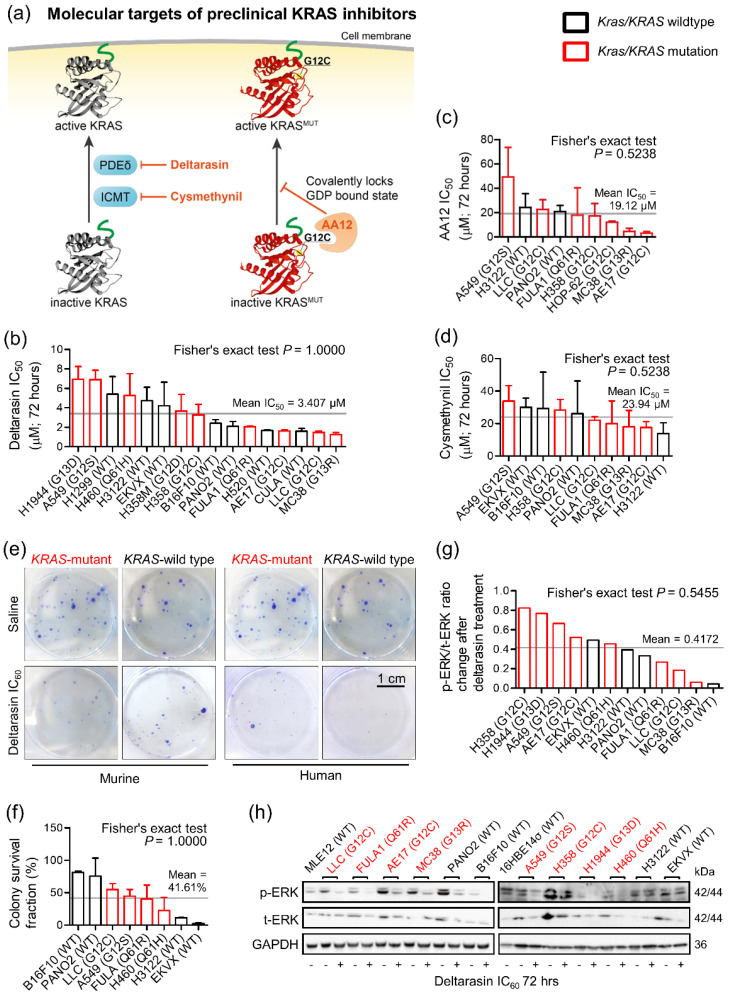
Pharmacologic evidence for *KRAS* mutation-independence in vitro. Different mouse and human tumor cell lines with (red) and without (black) *Kras/KRAS* mutations (codon changes are given in parentheses) were assessed for cell viability via colorimetric WST-8-assay, for colony formation via crystal violet-stained colony counts, and for ERK phosphorylation via phospho (p)- and total (t)-ERK immunoblots after 72-h treatments with three different KRAS inhibitors (*n* = 3/data-point). (**a**) Graphical scheme displaying molecular targets of preclinical KRAS inhibitors AA12, cysmethynil, and deltarasin. 3D protein structures obtained from RCSB PDB: human KRAS^WT^ (5VQ8, black), human KRAS^G12C^ (4LDJ, red). (**b**–**d**) IC_50_ of deltarasin (**b**), AA12 (**c**), and cysmethynil (**d**) as assessed via WST-8 assay. (**e**,**f**) Representative images of colonies after saline or IC_60_ deltarasin treatment (**e**) and colony survival fraction (**f**) after IC_60_ deltarasin, normalized to saline treatment. (**g**,**h**) Quantification of normalized p-ERK/t-ERK signal change after IC_60_ deltarasin, normalized to GAPDH (**g**) and representative immunoblots (**h**). (**b**–**d**,**f**,**g**) Data presented as mean ± SD. Grey lines represent the mean of all cell lines tested, which was used to dichotomize cell lines into sensitive and resistant. *p*, probability according to Fisher’s exact test for cross-tabulation of *Kras/KRAS* mutation status to drug sensitivity/resistance. KRAS, KRAS proto-oncogene GTPase; WT, wild-type; GAPDH, glyceraldehyde 3-phosphate dehydrogenase; ERK, extracellular-signal regulated kinase.

We initially investigated the cellular responses of a battery of human and murine cell lines with known *KRAS/Kras* (KRAS proto-oncogene, GTPase) mutation status [4,15,16,17] (Supplementary Figure S1a,b) to three preclinical KRAS inhibitors: deltarasin, targeting PDEδ (phosphodiesterase-δ) [7], AA12, allosterically targeting KRAS^G12C^ [8], and cysmethynil, targeting ICMT (isoprenylcysteine carboxylmethyltransferase) [6] (Figure 1a). For this, widely used assays were employed based on literature searches (Figure S1c). Initially, IC_50_ values were calculated from WST-8 assays performed after 72 h of treatment with half-log-incremental drug concentrations. Unexpectedly, all three KRAS inhibitors showed comparable efficacy across all cell lines tested, independent of their *KRAS/Kras* mutation status (Figure 1b–d and Supplementary Figure S2). Importantly, overall in vitro efficacy of all three drugs was modest, with IC_50_ values between 1–50 µM (Supplementary Tables S3–S5), while deltarasin had the lowest IC_50_ value. A literature search revealed that this was generally true for developmental KRAS inhibitors compared with tyrosine kinase inhibitors, which are effective at drug doses under 100 nM (Figure S1d).

To extend these results, we analyzed the response of eight selected murine and human cell lines to IC_60_ concentrations of deltarasin in an in vitro colony formation assay. Again, deltarasin efficacy was independent of *KRAS/Kras* mutation status (Figure 1e,f; Supplementary Figure S3). Since KRAS activates the mitogen-activated protein kinase cascade, inducing phosphorylation of ERK (extracellular-signal-regulated kinase), we quantified t- and p-ERK relative to GAPDH (glyceraldehyde-3-phosphate dehydrogenase) in 12 murine and human cell lines treated with saline or IC_60_ deltarasin. In line with the above results, deltarasin inhibited p-ERK independent of cellular *KRAS/Kras* mutation status (Figure 1g,h; Supplementary Figure S4).

Thus, pharmacologic KRAS inhibition did not reveal KRAS-dependence in vitro.

### 3.2. Specific In Vivo Effects of Deltarasin against KRAS-Mutant Tumors

**Figure 2:**
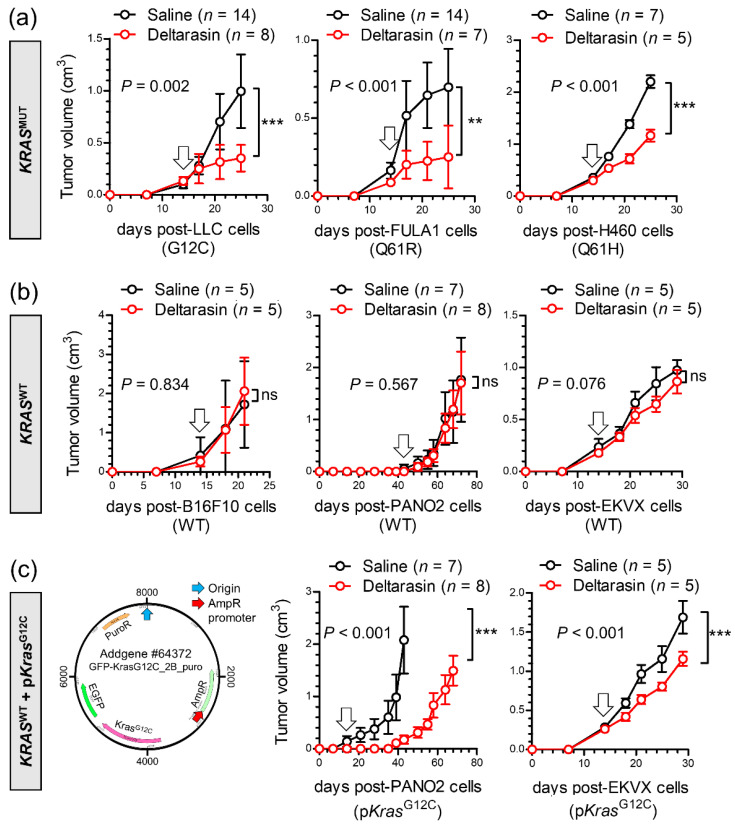
Deltarasin-mediated demonstration of *KRAS* mutation-dependence in vivo. Different mouse and human tumor cell lines with ((**a**); *KRAS*^MUT^) and without ((**b**); *KRAS*^WT^) endogenous *Kras/KRAS* mutations (codon changes are given in parentheses), as well as *KRAS*^WT^ cell lines forcedly expressing a plasmid encoding mutant murine *Kras*^G12C^ ((**c**); p*Kras*^G12C^), were injected into the rear flank (10^6^ tumor cells sc) of *C57BL/6* (LLC, B16F10, and PANO2 cells), *FVB* (FULA1 cells), or *Rag2*^−/−^ (H460 and EKVX cells) mice. After tumor establishment (tumor volume > 100 mm^3^; arrows), mice were randomly allocated to daily ip treatments with 100 μL saline (black) or 15 mg/ Kg deltarasin in 100 μL saline (red). Tumor growth was assessed by measuring three vertical tumor dimensions. Data presented as mean ± SD. *n*, sample size; *p*, overall probability, two-way ANOVA; ns, **, and ***: *p* > 0.05, *p* < 0.01, and *p* < 0.001, respectively, Bonferroni post-test. Rag2, recombination activating gene 2; sc, subcutaneously; ip, intraperitoneal.

To replicate these results in vivo, we induced subcutaneous tumors in *C57BL/6*, *FVB*, and *Rag2*^−/−^ (recombination activating gene 2) mice using six different cancer cell lines and initiated daily intraperitoneal saline or deltarasin (15 mg/Kg in saline) treatments after tumor establishment (tumor volume ≥ 100 mm^3^; latency ≥ 14 days post-sc injection). We pursued the inclusion of the other inhibitors in animal triage, but were unfortunately not successful in obtaining approval, largely due to the past extensive testing of cysmethynil and the sparse existing safety data for AA12 compared to our extensive experience with deltarasin [15,16]. Interestingly, deltarasin selectively inhibited the subcutaneous growth of murine and human *KRAS*-mutant tumors (Figure 2a), but had no effect on *KRAS*-*WT* tumors (Figure 2b). Moreover, forced overexpression of p*Kras*^G12C^ in *KRAS*-*WT* mouse and human cancer cells accelerated tumor growth and restored the response to the drug (Figure 2c).

Taken together, these data show that deltarasin-mediated KRAS inhibition selectively halted the growth of *KRAS*-mutant cancer cells in vivo.

### 3.3. Genetic KRAS Manipulation Reveals Contrasting KRAS-Dependencies In Vitro and In Vivo

**Figure 3:**
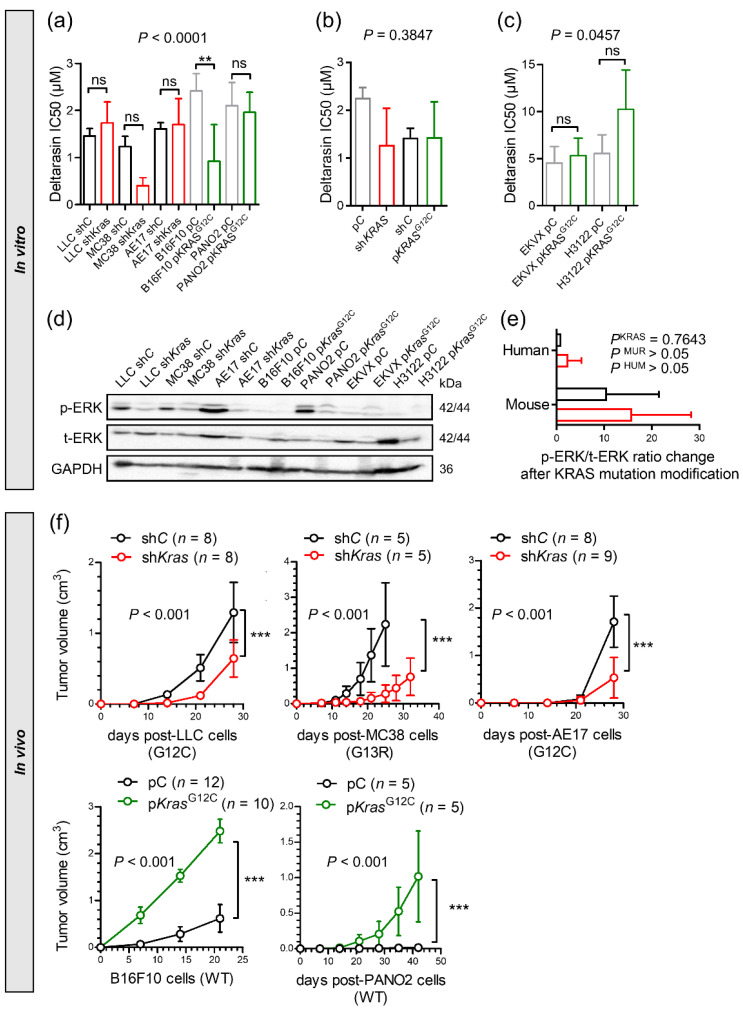
Genetic manipulation of *Kras* reveals in vivo-restricted KRAS dependence. (**a**) Different murine parental (black/grey: stably expressing random shRNA, sh*C*, or control plasmid, p*C*) or *Kras*-modified (red: stably expressing sh*Kras*; green: stably expressing mutant *Kras*^G12C^ plasmid, p*Kras*^G12C^) tumor cell lines were assessed for cell viability (IC_50_ via WST-8-assay; *n* = 2–4/data-point) after 72 h of deltarasin treatment. (**b**) Summary of averaged deltarasin IC_50_ values from all cell lines from (**a**) (*n* = 2–3 cell lines/group). (**c**) Human parental (black/grey: stably expressing control plasmid p*C*) or *KRAS*-modified (green: stably expressing p*Kras*^G12C^) tumor cell lines were assessed for cell viability via WST-8 assay (*n* = 2–5/data-point) after 72 h of deltarasin treatment. (**d**) Immunoblots of cell lines from (**a**) for p-ERK, t-ERK and GAPDH. (**e**) Quantification of normalized p-ERK/t-ERK signal from (**d**). Data were summarized by mutation status and origin. (**f**) The five cell line doublets from (**a**) were injected into the rear flank (10^6^ tumor cells sc) of *C57BL/6* mice for induction of flank tumors via genetically modified cells (red, sh*Kras*; green, p*Kras*^G12C^) or control cells (black, sh*C* or p*C*). *p*, overall probability according to one-way (**a**–**c**) and two-way (**e,f**) ANOVA. ns, **, and ***: *p* > 0.05, *p* < 0.01, and *p* < 0.001, respectively, for the indicated comparisons via Bonferroni post-tests. Data are presented as mean ± SD.

To further validate the observed in vivo-restricted specificity of deltarasin, we overexpressed anti-*Kras*-specific shRNA (sh*Kras*) in *Kras*-mutant parental cell lines or p*Kras*^G12C^ in *Kras-WT* parental cell lines [15]. In accordance with pharmacologic KRAS inhibition, genetic *Kras* modulation did not impact the in vitro response of cancer cell lines to deltarasin, as determined by WST-8 IC_50_ values and ERK activation levels (Figure 3a–e, Supplementary Figures S5 and S6). In contrast to the lack of *Kras*-dependence in vitro, mutant *Kras* was required and sufficient for sustained tumor growth in vivo (Figure 3f): murine cell lines expressing sh*Kras* displayed statistically (*p* < 0.001) and biologically (50–90% inhibition) significantly decreased tumor growth compared with parental cell lines expressing sh*C*. Correspondingly, p*Kras*^G12C^ overexpression accelerated tumor growth compared with overexpression of p*C*.

Collectively, these results supported that, similar to drug-based KRAS inhibition, genetic *Kras* modulation selectively impacts tumor growth in vivo.

### 3.4. A Mutant Kras Transcriptome Signature Contains Ccl2 and Il1b

**Figure 4:**
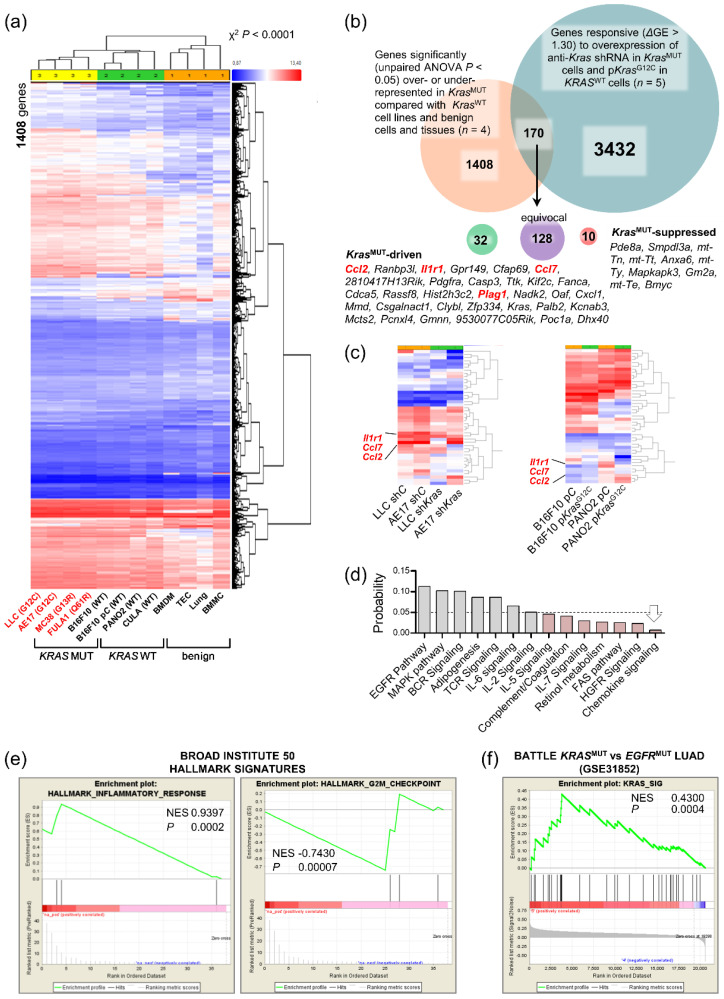
A 42-gene inflammatory signature of KRAS-dependence. (**a**) Unsupervised hierarchical clustering of gene expression of *Kras*-mutant and *Kras*-*WT* cancer cell lines, as well as benign cells and tissues. (**b**) Venn diagram of analytical strategy of transcriptome analysis. (**c**) Unsupervised hierarchical clustering of gene expression of *Kras*-modified cancer cell line doublets revealing co-clustering of *Il1r1* (interleukin-1 receptor 1) and *Ccl2* (C-C-motif chemokine ligand 2). (**d**) WikiPathway analysis showing pathways significantly overrepresented in the *KRAS* signature. (**e**) GSEA of 37 human orthologues of the murine *KRAS* signature against the Broad Institute’s 50 hallmark signatures, showing positive enrichment in the “inflammatory response” and negative enrichment in the “G2M checkpoint” signatures. NES, normalized enrichment score; *p*, family-wise error rate probability. (**f**) GSEA of 37 human orthologues of the murine *KRAS* signature against KRAS- (*n* = 21) versus EGFR-mutant (*n* = 17) lung adenocarcinomas (LUAD) from BATTLE revealing positive enrichment of our KRAS signature in human *KRAS*-mutant LUAD. NES, normalized enrichment score; *p*, family-wise error rate probability; EGFR, epidermal growth factor receptor.

In an effort to identify mutant-*Kras*-driven genes responsible for in vivo restricted KRAS-dependence, we analyzed the global transcriptomes of the parental and *Kras*-modulated murine cell lines described above and of benign samples (whole lungs, tracheal epithelial cells (TEC), and bone marrow-derived macrophages (BMDM) and mast cells (BMMC)). Unsupervised hierarchical clustering showed absolute segregation of benign, *Kras-WT*, and *Kras*-mutant samples by 1408 differentially expressed genes (ΔGE) using an ANOVA *p* < 0.05 threshold (Figure 4a,b). Paired analyses of the five isogenic cancer cell line doublets with modulated *Kras* (LLC, MC38, and AE17 cells expressing sh*C* versus sh*Kras* and PANO2 and B16F10 cells expressing p*C* versus p*Kras*^G12C^) identified another 3432 *Kras*-responsive transcripts. Out of the 170 transcripts that were present in both gene sets, 42 were both differentially represented in benign, *Kras-WT*, and *Kras*-mutant samples and responsive (ΔGE > 1.40) to *Kras* modulation, including *Kras* per se (Figure 4b, Supplementary Table S6). Interestingly, *Il1r1* (interleukin-1 receptor 1), *Ccl7* (C-C-motif chemokine ligand 7), and *Ccl2* were among those genes and were clustered tightly together (Figure 4c), and chemokine signaling was the pathway most significantly perturbed by *Kras* modulation on WikiPathway analysis (Figure 4d) [23].

We next translated our 42-gene murine mutant *Kras* signature to their 37 human orthologues using Orthoretriever (http://lighthouse.ucsf.edu/orthoretriever/; last accessed: 15 October.2021) and ran GSEA (http://software.broadinstitute.org/gsea/index.jsp; last accessed: 15 October 2021) [24]. Interestingly, our humanized mutant *KRAS* signature was enriched in only two of the Broad Institute’s 50 hallmark signatures: positively in the signature “inflammatory response” and negatively in the signature “G2M-checkpoint” (Figure 4e). Moreover, this mutant *KRAS* signature was significantly positively enriched in *KRAS*- versus *EGFR* (epidermal growth factor receptor)-mutant LUAD (lung adenocarcinoma; GSE43458) from the BATTLE trial [25,26] (Figure 4f). In connection with this, we recently reported that mutant *KRAS* drives CCL2 and Il1R1 expression in establishing inflammatory feedback loops with interleukin-1 beta (IL-1β)-secreting myeloid cells in malignant pleural effusions [15,16,20].

Collectively, the data further supported that in vivo-restricted mutant *KRAS*-dependence is mediated by proinflammatory signals to CCR2+ (C-C motif chemokine receptor) IL-1β-secreting host cells.

### 3.5. CCR2+ IL-1β-Secreting Myeloid Cells Potentiate In Vivo KRAS-Dependence

**Figure 5:**
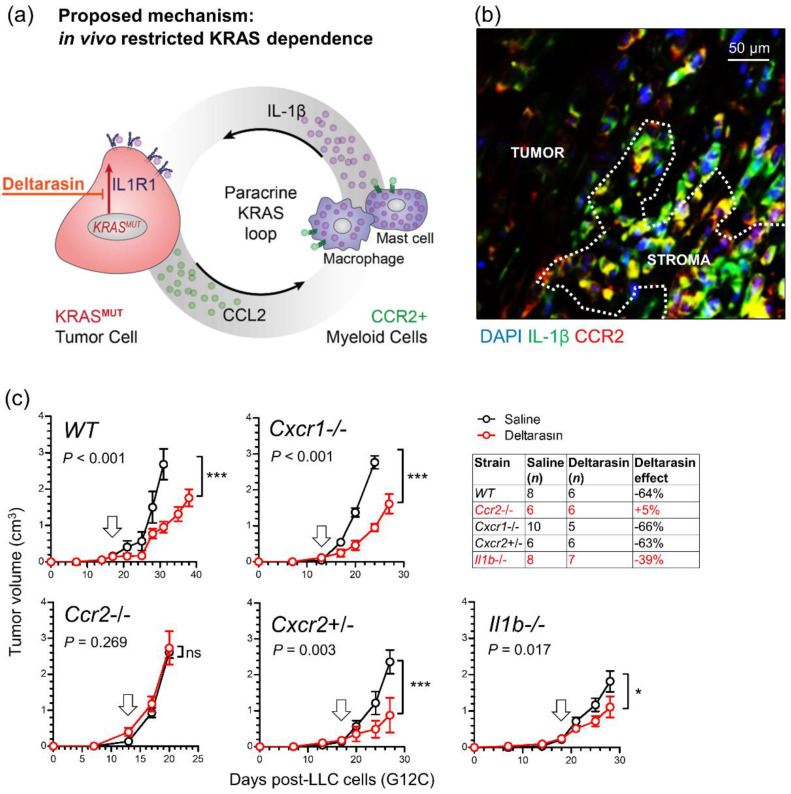
A requirement for host *Ccr2* and *IL1b* for KRAS dependence in vivo. (**a**) Graphical scheme of the proposed mechanism of in vivo restricted KRAS dependence. (**b**) Representative image of CCR2/IL-1β-co-staining of a *KRAS*-mutant tumor from a *Rag2*^−/−^ mouse showing co-localization of the two proteins in the tumor stroma. Image was taken using an AxioImager.M2 (Zeiss; Jena, Germany) with a 60× objective. (**c**) Syngeneic *C57BL/6* mice competent (*WT*) or deficient (*Il1b*^−/−^*, Ccr2*^−/−^) [18,20] in the *Il1b* and *Ccr2* genes or haplo/diplo-insufficient in the *Cxcr1* and *Cxcr2* chemokine receptor genes (*Cxcr1*^−/−^*, Cxcr2*^+/−^) received 10^6^ LLC cells (*Kras*^G12C^) sc followed by daily ip saline (black) or 15 mg/Kg deltarasin (red) treatments initiated when tumors reached 100 mm^3^ volumes (arrows). Data are presented as mean ± SD. *p*, overall probabilities according to two-way ANOVA; ns, *, and ***: *p* > 0.05, *p* < 0.05, and *p* < 0.001 for the indicated comparisons via Bonferroni post-tests. Table shows animal numbers used and percentile tumor inhibition by deltarasin compared with saline. *Ccr2*, C-C motif chemokine receptor; IL-1β, interleukin-1 beta; *Cxcr1/Cxcr2*, C-X-C motif chemokine receptor 1/2.

**Figure 6:**
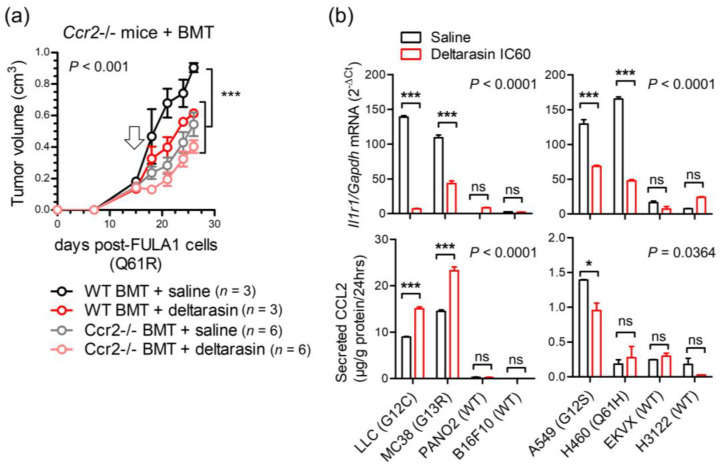
In vivo KRAS-dependence requires myeloid *Ccr2* and is abolished by deltarasin treatment via downregulation of *IL1R1* expression in *KRAS*-mutant cancer cells. (**a**) Total-body-irradiated (900 Rad) *Ccr2*^−/−^ mice received adoptive BMT from *WT* or *Ccr2*^−/−^ donors (all back-crossed > F12 to the *FVB* strain). After one month allowed for chimeric bone marrow reconstitution, chimeras received 10^6^ syngeneic FULA1 cells (*Kras*^Q61R^) sc [19]. Daily ip saline or deltarasin (15 mg/Kg in saline) treatments were started when tumors > 100 mm^3^ were established (arrow). Data are presented as mean ± SD. *p*, overall probabilities according to two-way ANOVA; ***: *p* < 0.001 for the indicated comparisons via Bonferroni post-tests. (**b**) *Il1r1/IL1R1* mRNA expression via qPCR (top) and CCL2 protein secretion via ELISA (bottom) of mouse (left) and human (right) cancer cell lines treated with saline or deltarasin IC_60_ for 72 h. Data are presented as mean ± SD. *p*, overall probabilities according to two-way ANOVA; ns, *, and ***: *p* > 0.05, *p* < 0.05 and *p* < 0.001, respectively, for the indicated comparisons via Bonferroni post-tests.

These results led us to the hypothesis that CCR2+ IL-1β-secreting myeloid cells are required for in vivo KRAS-dependence (Figure 5a). Indeed, numerous such cells co-expressing CCR2 and IL-1β were identified in the stromata of our experimental *Kras*-mutant tumors using immunohistochemistry (Figure 5b). To definitively test our hypothesis, we induced flank tumors by injecting 10^6^ LLC cells (*Kras*^G12C^) subcutaneously into syngeneic *C57BL/6* mice competent (*WT*) or deficient (*Il1b*^−/−^*, Ccr2*^−/−^) [18,20] in the *Il1b* and *Ccr2* genes. Mice haplo/diplo-insufficient in the *Cxcr1* (C-X-C motif chemokine receptor 1) and *Cxcr2* chemokine receptor genes (*Cxcr1*^−/−^*, Cxcr2*^+/−^) [17] were employed as additional controls for *Ccr2*^−/−^ mice and daily intraperitoneal saline or 15 mg/Kg deltarasin treatments were initiated when tumors reached 100 mm^3^ volumes. Expectedly, deltarasin treatment statistically and biologically significantly inhibited LLC tumor growth in *WT*, *Cxcr1*^−/−^, and *Cxcr2*^+/−^ mice. However, deltarasin effects were diminished in *Il1b*^−/−^ and completely abrogated in *Ccr2*^−/−^ mice (Figure 5c). To exclude the possibility of developmental effects in knockout mice, we total-body-irradiated (900 Rad) *Ccr2*^−/−^ mice and performed adoptive bone marrow transplants (BMT) from *WT* or *Ccr2*^−/−^ donors, as described previously [16,20]. For this experiment, *WT* and *Ccr2*^−/−^ mice back-crossed > F12 to the *FVB* strain were used together with syngeneic FULA1 cells (*Kras*^Q61R^) to obtain results with another cell line harboring a different *Kras* mutation and a broad mutation spectrum relevant to human *KRAS*-mutant LUAD [19]. Again, daily intraperitoneal saline or deltarasin treatments were started when tumors > 100 mm^3^ were established. Expectedly, *Ccr2*^−/−^ chimeras receiving *Ccr2*^−/−^ BMT did not respond to deltarasin, but *Ccr2*^−/−^ chimeras receiving *WT* BMT displayed markedly increased tumor growth as well as statistically and biologically significant inhibition from deltarasin treatment (Figure 6a). Collectively, these results indicated that myeloid CCR2 and IL-1β are required for deltarasin efficacy against *Kras*-mutant tumors in vivo.

### 3.6. Deltarasin Limits IL-1β Sensing by KRAS-Mutant Tumor Cells

We next interrogated the mechanism of in vivo-restricted deltarasin dependence. Based on the microarray-derived mutant *Kras* signature that encompassed *Ccl2* and *Il1r1* (Figure 4) and our previous reports on mutant *KRAS*-mediated transcriptional regulation of *CCL2* and *IL1R1* [15,16], we tested whether deltarasin blocks expression of these two genes (Figure 6b). Indeed, *KRAS*-mutant mouse and human cancer cell lines displayed markedly increased baseline *Il1r1/IL1R1* mRNA expression compared with *WT* cell lines, and significantly downregulated *Il1r1/IL1R1* transcript levels after deltarasin treatment (Figure 6b, upper panel). By comparison, only some *KRAS*-mutant cell lines displayed increased baseline CCL2 protein secretion compared with *WT* cell lines, and CCL2 elaboration was not consistently blocked by deltarasin treatment (Figure 6b, lower panel), suggesting that deltarasin-mediated downregulation of *Il1r1/IL1R1* expression delivered the bulk of the drug’s in vivo effects (Figure 5a).

### 3.7. An Inflammatory CCL2/IL1B Signature in KRAS-Mutant Human Cancers

**Figure 7:**
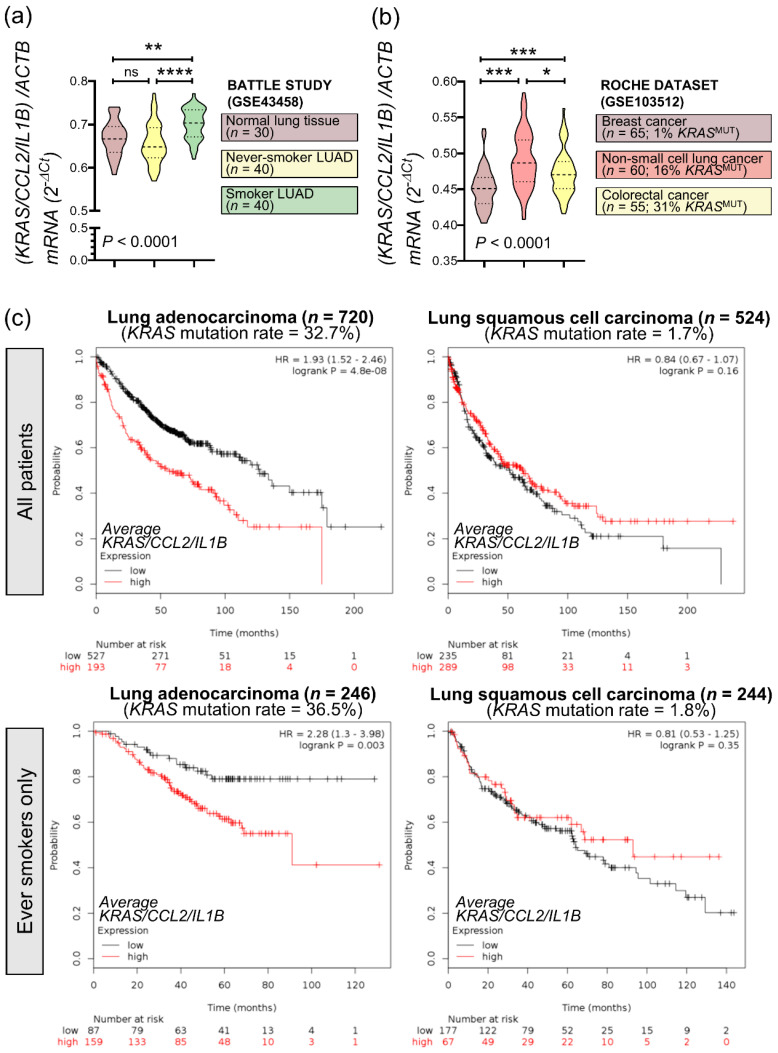
Mean expression of *KRAS/CCL2/IL1B* is increased in *KRAS*-mutant cancers and predicts poor survival. (**a**) Average *KRAS/CCL2/IL1B* expression normalized to *ACTB* in lung adenocarcinomas (LUAD) from smokers and never-smokers and normal lung tissue from never-smokers from the BATTLE study (GSE43458) [25,26]. (**b**) *KRAS/CCL2/IL1B* expression normalized to *ACTB* in breast, non-small cell lung, and colorectal cancer (ROCHE study GSE103512). *KRAS* mutation frequencies of these tumor types are from COSMIC [4]. (**c**) Kaplan-Meier analyses of lung cancer patients stratified by average *KRAS/CCL2/IL1B* expression performed using http://www.kmplot.com, accessed on 15 October 2021 [21]. *KRAS* mutation frequencies are from the Campbell cohort [27]. Top: all patients; Bottom: ever-smokers only. (**a**,**b**) Data are presented as violin plots. *p*, overall probability according to one-way ANOVA. ns, *, **, and ***: *p* > 0.05, *p* < 0.05, *p* < 0.01, and *p* < 0.001, respectively, for the indicated comparisons via Bonferroni post-tests. *ACTB*, beta-actin.

To investigate the relevance of our findings to *KRAS*-mutant human cancers, we analyzed the average expression of *KRAS*, *CCL2*, and *IL1B* genes in public data (GSE43458) from the BATTLE trial [25,26]. Interestingly, mean *KRAS/CCL2/IL1B* expression was statistically significantly increased in smokers’ LUAD (*n* = 40) compared with never-smokers’ LUAD (*n* = 40) and normal lung tissue samples (*n* = 30) (Figure 7a). Since *KRAS* mutations are more frequent in the LUAD of smokers [27], this finding suggested that our inflammatory signature was overrepresented in tumors with higher *KRAS* mutation frequencies. This was also true in another dataset from patients with breast, colorectal, and lung cancer (GSE103512) [28], where mean *KRAS/CCL2/IL1B* expression was significantly higher in lung and colorectal cancer, which have higher *KRAS* mutation rates [4], compared with breast cancer (Figure 7b).

Finally, online Kaplan-Meier analyses (http://www.kmplot.com, accessed on 15 October 2021) [21] using lung cancer patient data were performed (Figure 7c). These revealed that in patients with LUAD (a tumor with high KRAS mutation frequency), high *KRAS/CCL2/IL1B* expression levels portended 93% increased odds of death regardless of smoking status (Figure 7c, upper left). By contrast, *KRAS/CCL2/IL1B* expression did not impact the survival of patients with squamous cell lung carcinoma (a tumor with low KRAS mutation frequency) (Figure 7c, upper right). When exclusively smokers were examined (thereby enriching the sample for *KRAS*-mutant patients), high *KRAS/CCL2/IL1B* expression levels portended 128% increased odds of death in LUAD (Figure 7c, lower left) and continued to have no impact on the survival of patients with squamous cell lung carcinoma (Figure 7c, lower right).

Taken together, these data suggested that *KRAS/CCL2/IL1B* transcripts are overexpressed in human *KRAS*-mutant cancers and detrimentally affect survival. Importantly, the proposed KRAS-driven inflammatory loop may be clinically relevant.

## 4. Discussion

We hypothesized that mutant *KRAS* (KRAS proto-oncogene, GTPase) dependence occurs non-cell-autonomously and that KRAS inhibitor effects are delivered in vivo. We used 30 cancer cell lines with different *KRAS* mutations and multiple in vitro assays to show that both pharmacologic and genetic KRAS inhibition is selectively effective against *KRAS*-mutant murine and human tumors in vivo. Using isogenic cell lines with intact or compromised mutant *KRAS* signaling, we identified a novel *KRAS*-mutation-specific transcriptome signature that is surprisingly dominated by inflammatory response genes, including *CCL2* (C-C-motif chemokine ligand 2) and *IL1B* (interleukin-1 beta, IL-1β). We further employed several transgenic mouse strains and adoptive bone marrow transfer experiments to show that effective pharmacologic KRAS blockade in vivo is dependent on the presence of CCR2+ (C-C motif chemokine receptor 2) IL-1β-secreting myeloid cells in the tumor microenvironment. Finally, we showed that the KRAS blocker deltarasin acts to downregulate *IL1R1* (interleukin-1 receptor 1) expression in *KRAS*-mutant tumor cells and that the proposed *KRAS/CCL2/IL1B* signature is enriched in human cancers with high *KRAS* mutation frequencies, in which it portends a dismal prognosis. Our results imply that conventional cell-based screens for the discovery and development of novel KRAS blockers might be suboptimal, and that IL-1β inhibition may be specifically effective against KRAS-mutant cancers.

A long line of evidence supports that homotypic two-dimensional cancer cell cultures are not optimal for the study of KRAS-dependence. Singh et al. established a “RAS-dependency index” in a large panel of human lung and pancreatic cancer cell lines, systematically addressing the variable of in vitro efficacy of KRAS inhibition [12]. Project DRIVE, a comprehensive synthetic lethality screen applying > 150,000 shRNAs on 7837 genes and 398 cancer cell lines (https://oncologynibr.shinyapps.io/drive/, accessed on 15 October 2021), identified no lethal interaction partners for KRAS in vitro, a finding that urged the authors to state: “… the data here raise the likelihood that no single synthetic lethal gene will be found across all KRAS mutant tumors … commonly used KRAS mutant models are not KRAS dependent, when interrogated as monolayer cell cultures … ablating KRAS dependence will need to carefully consider these findings …” [14]. Recently, Janes et al. developed ARS-1620, a new covalent G12C-specific KRAS inhibitor that is highly effective in vivo, but not in vitro [9]. The authors developed three-dimensional co-culture systems and state: “We use ARS-1620 to dissect oncogenic KRAS dependency and demonstrate that monolayer culture formats significantly underestimate KRAS dependency in vivo”. Despite the tremendous progress contributed by the above-referenced work, the mechanism(s) of the observed in vivo-restricted KRAS-dependence remained obscure prior to this report.

To this end, multiple lines of work support the notion that the paracrine effects of KRAS and other RAS oncogenes overshadow their cell-autonomous impact. A pioneering report identified how RAS oncogenes utilize paracrine IL-8 signaling to induce angiogenesis in vivo [13,29]. We determined how *KRAS*-mutant cancer cells depend on paracrine CCL2 signaling to myeloid cells, including mononuclear and mast cells, to induce vascular permeability and angiogenesis during malignant pleural effusion development [15,20]. In turn, myeloid-derived IL-1β was found to selectively trigger non-canonical nuclear factor (NF)-κΒ activation in *KRAS*-mutant cancer cells via IL1R1 and inhibitor of NF-κΒ kinase α (ΙΚΚα), with the latter presenting a marked therapeutic target in mouse models of pre-metastatic and advanced lung cancer [16,30]. Here, we showed how deltarasin functions to abrogate a mutant *KRAS*-initiated in vivo inflammatory loop of tumor-derived CCL2 and myeloid-secreted IL-1β by downregulating the IL1R1 expression of *KRAS*-mutant tumor cells and thereby abolishing their receptivity to myeloid IL-1β signals. We identified CCR2+ myeloid cells that provide IL-1β to the microenvironment of *KRAS*-mutant tumors and showed that they are required for mutant *KRAS* dependence in vivo. Data from syngeneic mouse models of global host *Ccr2* and *Il1b* gene deficiency and of focal myeloid *Ccr2* reconstitution are further supported by human cancer xenograft experiments in *Rag2*^−/−^ (recombination activating gene 2) mice, which lack B- and T-cell function but feature intact myeloid cells [31], to collectively identify the proposed inflammatory loop that potentiates KRAS blockade. The growing evidence that KRAS is a key modulator of the inflammatory tumor microenvironment and immune escape was recently extensively reviewed by Hamarsheh and colleagues [32]. Interestingly, recent studies also indicate the association between KRAS mutation and anti-tumor immunity. For instance, the first approved KRAS^G12C^ inhibitor, sotorasib (AMG510), resulted in a pro-inflammatory tumor microenvironment in immune-compentent mice and anti-tumor activity in clinical trials [33], while oncogenic KRAS^G12D^ promotes a pro-inflammatory RAC1 (rac family small GTPase 1)/ROS (reactive oxygen species)/NLRP3 (NLR family pyrin domain containing 3)/IL-1β axis additionally to its canonical oncogenic driver function [34].

In addition to *Kras*, *Ccl2*, and *Il1b*, a battery of other transcripts originated within the signature of *KRAS*-mutant cancers derived from the transcriptomes of our cell lines, providing synthetic lethality candidates for in vivo KRAS dependency for future research. This signature includes signal transducers *Ranbp3l* (RAN binding protein 3 like), *Gpr149* (G protein-coupled receptor 149), and *Rassf8* (Ras association domain family member 8), inflammatory messengers *Ccl7*, *Cxcl1* (C-X-C motif chemokine ligand 1), and *Casp3* (caspase 3), cell surface receptors *Pdgfra* (platelet derived growth factor receptor alpha) and *Ttk* (TTK protein kinase), and cell cycle genes and tumor suppressors *Cdca5* (cell division cycle associated 5), *Hist2h3c2* (histone cluster 2 H3 family member C2), *Plag1* (pleomorphic adenoma gene 1), *Fanca* (FA complementation group A), and *Gmnn* (geminin DNA replication inhibitor), among others. The importance of some of these candidates is worth mentioning: *Cxcl1* was recently found to mediate the effects of KRAS-IKKα addiction during malignant pleural effusion development [16]; *Casp3* is a central effector of compensatory tumor proliferation and radiotherapy resistance [35]; and *Gmnn* was recently found to function as a tumor suppressor in lung and colon cancer [36]. Surprisingly, *Kras* mutation status imprinted the transcriptomes of our cell lines more profoundly than their tissues of origin, causing them to cluster together in an unsupervised fashion. Furthermore, our *KRAS*-mutation signature was enriched in human *KRAS*-mutant cancers and predicted poor survival, a fact that further validates this gene set. Most importantly, the mutant *KRAS* signature was dominated by the inflammatory response pathway according to both WikiPathways analysis and GSEA, highlighting the notion that the oncogene functions in a proinflammatory fashion.

In addition to fostering the battle to drug KRAS, the present work bears significant clinical implications by pinning CCL2 and IL-1β as key inflammatory addiction partners of mutant *KRAS*. Although targeting CCL2 with neutralizing antibodies yielded promising preclinical results [15,20,37,38,39,40], clinical trials of the anti-human CCL2 antibody carlumab were hampered by limited drug efficacy and tolerability [41,42,43]. In contrast, targeting IL-1β with canakinumab has raised enthusiasm and holds great promise in cancer therapy. In this regard, the Canakinumab Anti-inflammatory Thrombosis Outcomes Study (CANTOS), a randomized trial on the role of IL-1β inhibition in atherosclerosis, secondarily aimed at establishing whether low (50 mg), medium (150 mg), or high (300 mg)-dose canakinumab given sc every three months might alter cancer incidence [44,45]. The results astonished, with total cancer mortality decreasing by 51% in the high-dose group, incident lung cancer decreasing by 39% in the medium-dose and by 67% in the high-dose groups, and lung cancer mortality decreasing by 77% in the high-dose group. Although our results showing diminished deltarasin efficacy with *Il1b*^−/−^ mice were less impressive compared with the complete abrogation of deltarasin effects in *Ccl2*^−/−^ mice, we believe that this is attributable to redundant IL-1α (interleukin-1 alpha) signaling in the former and that targeting IL-1β might be specifically effective against *KRAS*-mutant cancers [46,47,48,49,50]. This is plausible according to CANTOS results, since canakinumab effects in decreasing lung cancer incidence and mortality were double in current than in past smokers overall, and quadruple when the high-dose group was examined alone, with current smokers having higher *KRAS* mutation rates than never-smokers [4,25,26,27]. Our results suggest that canakinumab might be selectively effective against *KRAS*-mutant cancers and warrant an a posteriori analysis of CANTOS results with respect to *KRAS* mutation status. In addition, the inflammatory loop described herein needs to be tested and validated in a new molecular subtype of malignant pleural mesothelioma we recently discovered [51].

## 5. Conclusions

In summary, we showed that *KRAS*-mutant cancer cells express CCL2 and IL1R1 to initiate an inflammatory signaling loop with CCR2/IL-1β-expressing myeloid cells. Our work indicated that this crosstalk is required for KRAS-dependence and blockade, which targets IL1R1 expression. The data set a rational framework for the future development of effective KRAS inhibitors and design of clinical trials aimed at targeting IL-1β in cancer.

## Data Availability

All microarray data have been deposited at GEO (http://www.ncbi.nlm.nih.gov/geo/, Last accessed: 15 October 2021; Accession ID: GSE130624) and are accessible using the link https://www.ncbi.nlm.nih.gov/geo/query/acc.cgi?acc=GSE130624, Last accessed: 15 October 2021, (murine microarrays). Published studies that were re-analyzed are freely accessible using the links: https://www.ncbi.nlm.nih.gov/geo/query/acc.cgi?acc=GSE43458, Last accessed: 15 October 2021, https://www.ncbi.nlm.nih.gov/geo/query/acc.cgi?acc=GSE31852, Last accessed: 15 October 2021 and https://www.ncbi.nlm.nih.gov/geo/query/acc.cgi?acc=GSE103512, Last accessed: 15 October 2021.

## Supplementary Materials

The following are available online at https://www.mdpi.com/article/10.3390/biomedicines10030592/s1, Figure S1: Mutation status of cell lines used in this study, in vitro assays used in cancer research and comparative efficacy of KRAS versus tyrosine kinase inhibitors, Figure S2: Response of *KRAS*-mutant tumor cells to *KRAS* inhibitors analyzed via WST-1 assay, Figure S3: Response of *KRAS*-mutant tumor cells to *KRAS* inhibitors analyzed via colony formation assay, Figure S4: Uncropped blots for Figure 1h, Figure S5: Validation of p*Kras*^G12C^ transduction in human cell lines H3122 and EKVX, Figure S6: Uncropped blots for Figure 3d; Table S1: Antibodies used in this study, Table S2: Oligonucleotides for qPCR, Table S3: Deltarasin effects on a battery of murine and human cancer cell lines, Table S4: AA12 effects on a battery of murine and human cancer cell lines, Table S5: Cysmethynil effects on a battery of murine and human cancer cell lines, Table S6: A 42-gene mutant *KRAS* signature identified from microarray analyses.
