# Supplementary material for: An In Vivo Inflammatory Loop Potentiates KRAS Blockade
Source: Biomedicines. 2022 Mar 3;10(3):592. doi: 10.3390/biomedicines10030592 (PMC8945202; doi:10.3390/biomedicines10030592)
Supplement: Supplementary file 1 [file biomedicines-10-00592-s001.zip › biomedicines-1596404-supplementary.pdf]

SUPPLEMENTARY INFORMATION

An *in vivo* inflammatory loop potentiates KRAS blockade

Kristina A.M. Arendt[§], Giannoula Ntaliarda[§], Vasileios Armenis, Danai Kati, Christin Henning, Georgia A. Giotopoulou, Mario A.A. Pepe, Laura V. Klotz, Anne-Sophie Lamort, Rudolf A. Hatz, Sebastian Kobold, Andrea C. Schamberger, and Georgios T. Stathopoulos.

SUPPLEMENTARY TABLES

Supplementary Table S1. Antibodies used in this study.

Method	Target protein	Provider	Catalog number	Dilution
WB	p-ERK	Santa Cruz Biotechnology	sc-7383	1:1000
WB	t-ERK	Santa Cruz Biotechnology	sc-514302	1:1000
WB	GAPDH	Cell Signaling	#2118	1:2000
WB	rat anti-mouse IgG	Abcam	ab131368	1:10000
WB	anti-rabbit IgG VHH	Abcam	ab191866	1:10000
IF	IL-1ß-Alexa488	Santa Cruz Biotechnology	sc-5155988 AF488	1:50
IF	CCR2	Thermo Fisher Scientific	PA5-23043	1:50
IF	donkey anti-rabbit IgG AlexaFluor647	Abcam	ab150075	1:500
IF	normal mouse IgG2a Alexa Fluor488	Santa Cruz Biotechnology	sc-3891	1:50
IF	normal mouse IgG1 Alexa Fluor488	Santa Cruz Biotechnology	sc-3890	1:50

Supplementary Table S2. Oligonucleotides for qPCR.

**Primer**	**Sequence**	**Amplicon (bp)**
Murine *Il1r1*F	GCTGACTTGAGGCAGTT	200
Murine *Il1r1*R	CATACGTCAATCTCCAGCGAC	
Human *IL1R1*F	AGGTAGACGCACCCTCTGAA	154
Human *IL1R1*R	GCATTTATCAGCCTCCAGAGAAG	
Murine *Gapdh*F	CCCTTAAGAGGGATGCTGCC	124
Murine *Gapdh*R	TACGGCCAAATCCGTTCACA	
Human *GAPDH*F	TTAGGAAAGCCTGCCGGTGA	157
Human *GAPDH*R	GGCGCCCAATACGACCAAA	

SupplementaryTable S3.Deltarasin effects on a battery of murine and human cancer cell lines.

**Originating organism**	**Cell line**	**Tissue origin**	***KRAS* mutation**	**IC_{50} (µM, mean±SD)[a]**	***n***
C57BL/6 mouse	LLC	Lewis lung carcinoma	G12C	1.46 ± 0.16	3
C57BL/6 mouse	MC38	Colon adenocarcinoma	G13R	1.23 ± 0.22	3
C57BL/6 mouse	AE17	Malignat pleural mesothelioma	G12C	1.61 ± 0.13	3
FVB mouse	FULA	Urethane-induced lung adenocarcinoma	Q61R	2.10 ± 0.06	3
C57BL/6 mouse	B16F10	Malignant skin melanoma	None	2.41 ± 0.37	3
C57BL/6 mouse	PANO2	Pancreatic adenocarcinoma	None	2.10 ± 0.49	4
C57BL/6 mouse	CULA	Urethane-induced lung adenocarcinoma	None	1.59 ± 0.29	3
Human	A549	Lung adenocarcinoma	G12S	6.90 ± 0.96	3
Human	H460	Lung large cell carcinoma	Q61H	5.27 ± 2.24	3
Human	H358	NSCLC	G12C	3.27 ±1.10	3
Human	H358M	Bronchiolo-alveolar carcinoma	G12D	3.67 ± 1.70	3
Human	H1944	NSCLC	G13D	6.93 ± 1.32	3
Human	H520	Squamous cell carcinoma	None	1.67 ± 0.06	3
Human	EKVX	Lung adenocarcinoma	None	4.22 ± 2.41	3
Human	H1299	NSCLC	None	5.40 ± 1.81	3
Human	H3122	NSCLC	None	4.73 ± 1.38	3

[a] IC_{50}, 50% inhibitory concentration by WST-8 assay (Biotool); SD, standard deviation; *n*, sample size; NSCLC, non-small cell lung cancer.

Supplementary Table S4. AA12 effects on a battery of murine and human cancer cell lines.

**Originating organism**	**Cell line**	**Tissue origin**	***KRAS* mutation**	**IC_{50} (μM, mean±SD)[a]**	***n***
C57BL/6 mouse	LLC	Lewis lung carcinoma	G12C	22.69 ± 7.95	3
C57BL/6 mouse	MC38	Colon adenocarcinoma	G13R	4.59 ± 2.45	2
C57BL/6 mouse	AE17	Malignant pleural mesothelioma	G12C	3.06 ± 1.39	2
FVB mouse	FULA1	Urethane-induced lung adenocarcinoma	Q61R	4.97 ± 2.28	2
C57BL/6 mouse	PANO2	Pancreatic adenocarcinoma	None	20.85 ± 5.11	2
Human	A549	Lung adenocarcinoma	G12S	49.30 ± 24.31	3
Human	H358	NSCLC	G12C	27.85 ± 4.41	2
Human	H3122	NSCLC	None	24.24 ± 11.41	3

[a] IC_{50}, 50% inhibitory concentration by WST-1 assay (Biotool); SD, standard deviation; *n*, sample size; NSCLC, non-small cell lung cancer.

Supplementary Table S5. Cysmethynil effects on a battery of murine and human cancer cell lines.

**Originating organism**	**Cell line**	**Tissue origin**	***KRAS* mutation**	**IC_{50} (μM, mean±SD)[a]**	***n***
C57BL/6 mouse	LLC	Lewis lung carcinoma	G12C	22.11 ± 2.19	2
C57BL/6 mouse	MC38	Colon adenocarcinoma	G13R	18.13 ± 9.92	2
C57BL/6 mouse	AE17	Malignant pleural mesothelioma	G12C	17.62 ± 3.57	3
FVB mouse	FULA1	Urethane-induced lung adenocarcinoma	Q61R	27.37 ± 7.78	2
C57BL/6 mouse	B16F10	Malignant skin melanoma	None	19.61 ± 13.27	3
C57BL/6 mouse	PANO2	Pancreatic adenocarcinoma	None	16.92 ± 17.22	2
Human	A549	Lung adenocarcinoma	G12S	30.84 ± 9.10	3
Human	H358	NSCLC	G12C	28.32 ± 6.57	3
Human	EKVX	Lung adenocarcinoma	None	30.05 ± 5.66	3
Human	H3122	NSCLC	None	10.95 ± 3.77	3

[a] IC_{50}, 50% inhibitory concentration by WST-1 assay (Biotool); SD, standard deviation; *n*, sample size; NSCLC, non-small cell lung cancer.

Supplementary Table S6. A 42-gene mutant *KRAS* signature identified from microarray analyses.

Genes significantly ($P < 0.05$ by unpaired ANOVA with Bonferroni post-tests) differentially represented in *KRAS*-mutant tumor cells compared with *KRAS*-wild-type tumor cells and benign cells and tissues, at the same time >30% responsive to modulation of *KRAS* expression in all five tumor cell line doublets tested (LLC, MC38, and AE17 cells expressing sh*C* versus sh*Kras* and PANO2 and B16F10 cells expressing p*C* versus p*Kras*^{G12C}).

Gene Symbol	Description	*Kras*^{WTa}	*Kras*^{MUTb}	% *Kras*^{Rc}
Ccl2	chemokine (C-C motif) ligand 2	0.82	37.79	56.29
Ranbp3l	RAN binding protein 3-like	0.93	35.26	78.33
Il1r1	interleukin 1 receptor, type I	2.39	25.63	36.71
Gpr149	G protein-coupled receptor 149	0.80	22.94	55.68
Cfap69	cilia and flagella associated protein 69	4.50	20.11	40.29
Ccl7	chemokine (C-C motif) ligand 7	0.96	16.11	50.14
2810417H13Rik	RIKEN cDNA 2810417H13 gene	11.39	12.64	42.33
Pdgfra	platelet derived growth factor receptor α	1.89	12.38	41.60
Casp3	caspase 3	4.20	10.48	30.74
Ttk	Ttk protein kinase	7.94	9.58	45.13
Kif2c	kinesin family member 2C	5.98	7.78	45.06
Fanca	Fanconi anemia, complementation group A	4.00	5.58	46.78
Cdca5	cell division cycle associated 5	3.56	5.43	47.15
Rassf8	Ras association (RalGDS/AF-6) domain family (N-terminal) member 8	2.46	5.35	32.08
Hist2h3c2	histone cluster 2, H3c2	1.20	4.69	38.87
Plag1	pleiomorphic adenoma gene 1	0.78	4.53	53.15
Nadk2	NAD kinase 2, mitochondrial	1.58	4.50	50.89
Oaf	OAF homolog (Drosophila)	2.39	4.23	31.03
Cxcl1	chemokine (C-X-C motif) ligand 1	1.56	4.23	73.09
Mmd	monocyte to macrophage differentiation-associated	2.93	4.06	35.92
Csgalnact1	chondroitin sulfate N-acetylgalactosaminyltransferase 1	0.74	3.97	50.96
Clybl	citrate lyase beta like	1.72	3.76	42.33
Zfp334	zinc finger protein 334	1.04	3.68	58.59
Kras	v-Ki-ras2 Kirsten rat sarcoma viral oncogene homolog	2.08	2.60	39.71
Palb2	partner and localizer of BRCA2	2.39	2.57	30.55
Kcnab3	potassium voltage-gated channel, shaker-related subfamily, beta member 3	1.25	2.55	49.23
Mcts2	malignant T cell amplified sequence 2	1.45	2.36	30.65
Pcnxl4	pecanex-like 4 (Drosophila)	1.38	2.19	39.54
Gmnn	geminin	1.39	2.04	34.57

9530077C05Rik	RIKEN cDNA 9530077C05 gene	1.17	1.88	30.46
Poc1a	POC1 centriolar protein homolog A	1.46	1.68	36.71
Dhx40	DEAH (Asp-Glu-Ala-His) box polypeptide 40	1.28	1.67	31.03
Pde8a	phosphodiesterase 8A	1.54	0.23	-181.67
mt-Tt	mitochondriallyencoded tRNA theonine	0.78	0.22	-95.34
Mapkapk3	mitogen-activated protein kinase-activated protein kinase 3	0.60	0.20	-96.43
Anxa6	annexin A6	0.69	0.19	-76.05
mt-Te	mitochondrially encoded tRNA glutamic acid	0.33	0.16	-49.69
mt-Ty	mitochondriallyencoded tRNA tyrosine	0.22	0.15	-77.77
Bmyc	brain expressed myelocytomatosis oncogene	1.42	0.15	-71.71
Gm2a	GM2 ganglioside activator protein	0.40	0.12	-70.05
Smpdl3a	sphingomyelin phosphodiesterase, acid-like 3A	1.21	0.11	-123.77
mt-Tn	mitochondriallyencoded tRNA asparagine	0.33	0.11	-105.91

[a] Normalized mRNA expression levels of *Kras*-wild-type (WT) cell lines (B16F10, B16F10 stably expressing p*C*, PANO2, and CULA cells) shown as fraction of expression of benign cells (bone marrow-derived macrophages and mast cells, tracheal epithelial cells) and lungs (*n* = 4/group).

[b] Normalized mRNA expression levels of *Kras*-mutant (MUT) cell lines (LLC, MC38, AE17, and FULA cells) shown as fraction of expression of benign cells (bone marrow-derived macrophages and mast cells, tracheal epithelial cells) and lungs (*n* = 4/group).

[c] Percentile mean response (R) of mRNA expression levels to *Kras* modulation, including *Kras* silencing of *Kras*-mutant cell lines (LLC, MC38, and AE17 cells) and overexpression of mutant $Kras^{G12C}$ plasmid in *Kras*-wild-type cells (B16F10 and PANO2 cells). Positive responses indicate suppression by *Kras* silencing and induction by overexpression of mutant $Kras^{G12C}$ plasmid. Negative responses indicate induction by *Kras* silencing and suppression by overexpression of mutant $Kras^{G12C}$ plasmid.

LLC, *C57BL/6* Lewis lung carcinoma; MC38, *C57BL/6* colon adenocarcinoma; AE17, *C57BL/6* malignant pleural mesothelioma; FULA, *FVB* urethane-induced lung adenocarcinoma; B16F10, *C57BL/6* malignant skin melanoma; PANO2, *C57BL/6* pancreatic adenocarcinoma; CULA, *C57BL/6* urethane-induced lung adenocarcinoma.

SUPPLEMENTARY FIGURES

Supplementary Figure S1

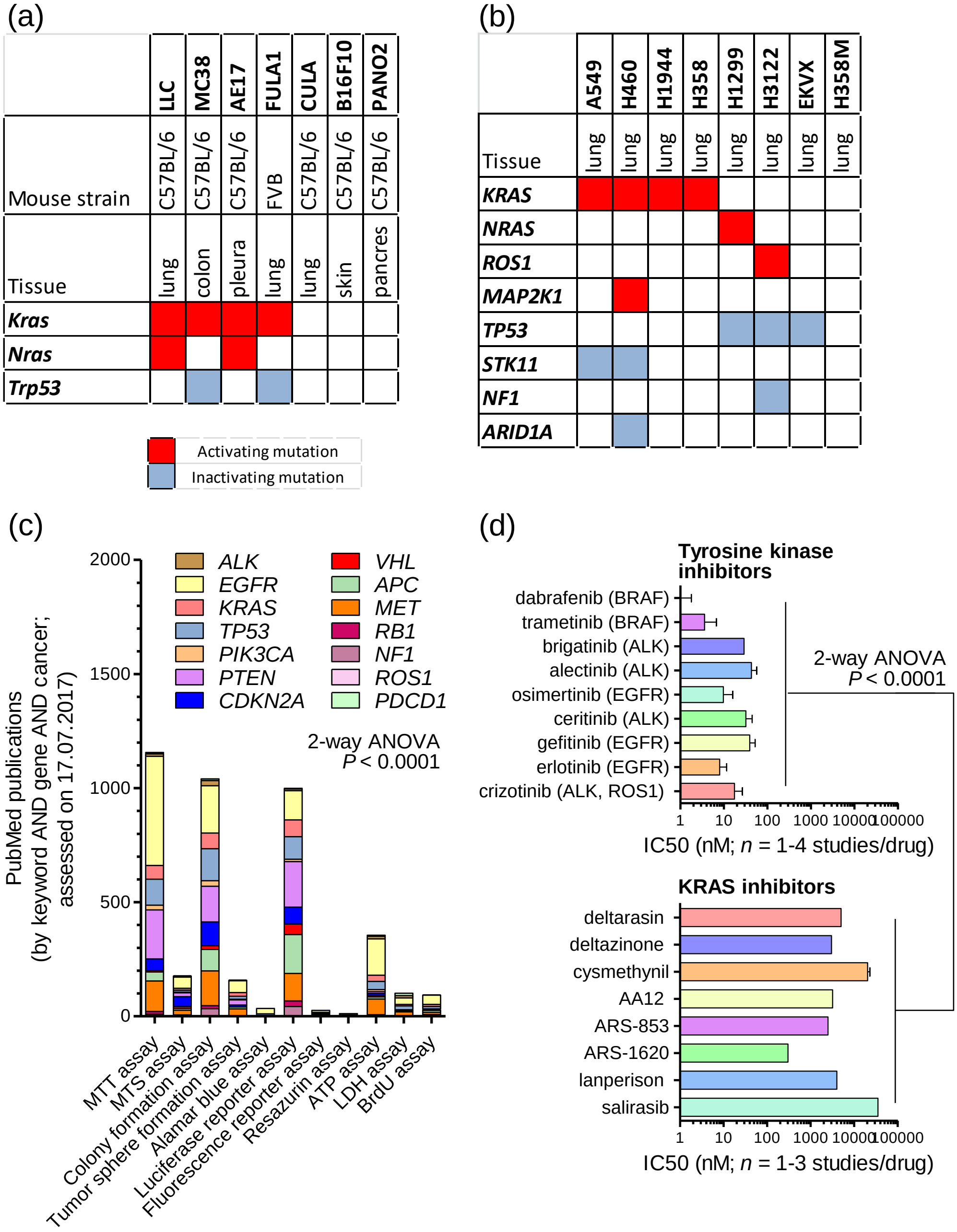

Figure S1. Mutation status of cell lines used in this study, *in vitro* assays used in cancer re-search and comparative efficacy of KRAS versus tyrosine kinase inhibitors.

(a) Murine cell lines used in this study with their syngeneic mouse strain, tissue of origin, and mutation status of *Kras, Nras*, and *Trp53*. Data from [S1-S6]. Red = activating mutations, blue = inactivating mutations.

(b) Human cell lines used in this study with their tissue of origin and mutation status of *KRAS*, *NRAS*, *ROS1*, *MAP2K1*, *TP53*, *STK11*, *NF1*, and *ARID1A*. Data from [S7]. Red = activating mu-tations, blue = inactivating mutations.

(c) Summary of *in vitro* assays used in cancer research stratified by target gene. Data are from a PubMed search done between 17-29.07.2018 using search strategy ("assay type" AND "gene" AND "cancer") and the number of retrieved publications as the readout. Assay types are listed in the x-axis and genes in the legend.MTT,3-(4,5-dimethylthiazol-2-yl)-2,5-diphenyltetrazolium bromide;MTS,3-(4,5-dimethylthiazol-2-yl)-5-(3-carboxymethoxyphenyl)-2-(4-sulfophenyl)-2H-tetrazolium); ATP, adenosine triphosphate; LDH, Lactate dehydrogenase; BrdU, bromodeoxyuri-dine, 5-bromo-2'-deoxyuridine.***P***, overall probability by 2-way ANOVA. Note that MTT/MTS and colony formation assays are the most commonly used and were used in this study.

(d) Fifty percent inhibitory concentrations (IC_{50}) of selected FDA-approved tyrosine kinase in-hibitors (TKI; top) and of published KRAS inhibitors (bottom) in preclinical development. *n*, published studies; *P*, overall probability by 2-way ANOVA. Note the statistically significantly higher and physiologically difficult to achieveIC_{50} of KRAS inhibitors compared with TKI. Data were from [S8-S25].

Supplementary Figure S2

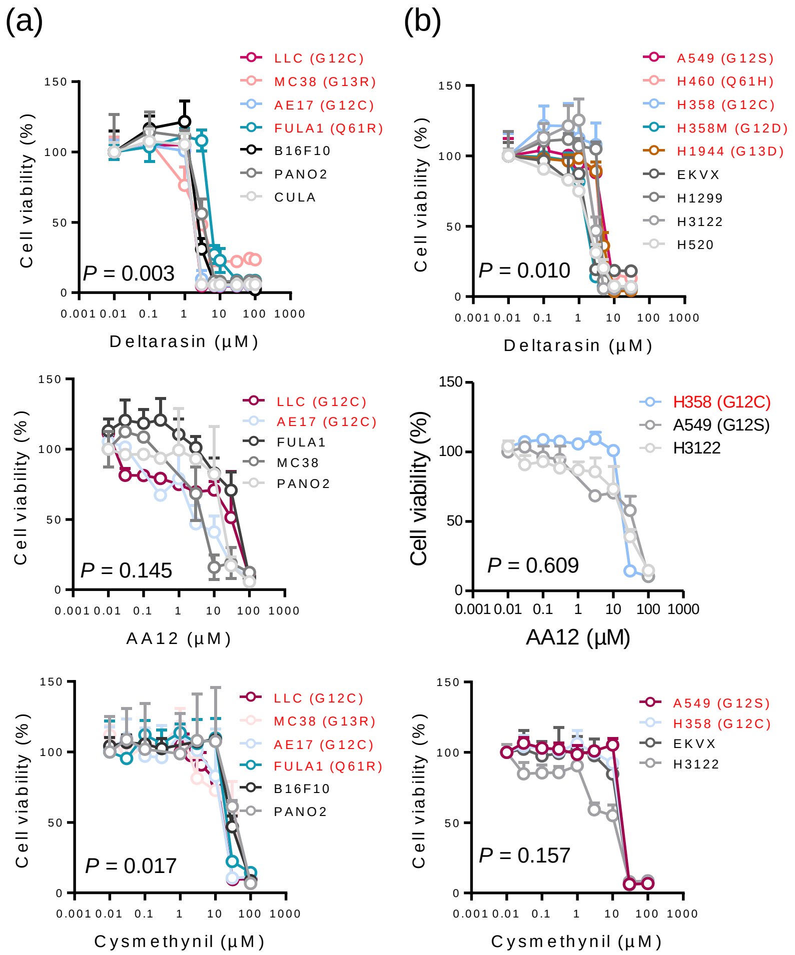

Figure S2. Response of *KRAS*-mutant tumor cells to *KRAS* inhibitors analyzed by WST-1 assay.

Different mouse (**a**; *Kras*^{MUT}: LLC, MC38, AE17, FULA1; *Kras*^{WT}: B16F10, CULA, PANO2) and human (**b**; *KRAS*^{MUT}: A549, H460, H358, H358M, H1944, HOP-62; *KRAS*^{WT}: EKVX, H1299, H3122, H520) tumor cell lines were assessed for inhibition of cell viability (determined by WST-1 assay, n = 3/data-point) by three different KRAS inhibitors: deltarasin (top), AA12 (middle), and cysmethynil (bottom).

Data presented as mean ± SD. P, overall probability by nonlinear fit and extra sum of squares F-test.

Supplementary Figure S3

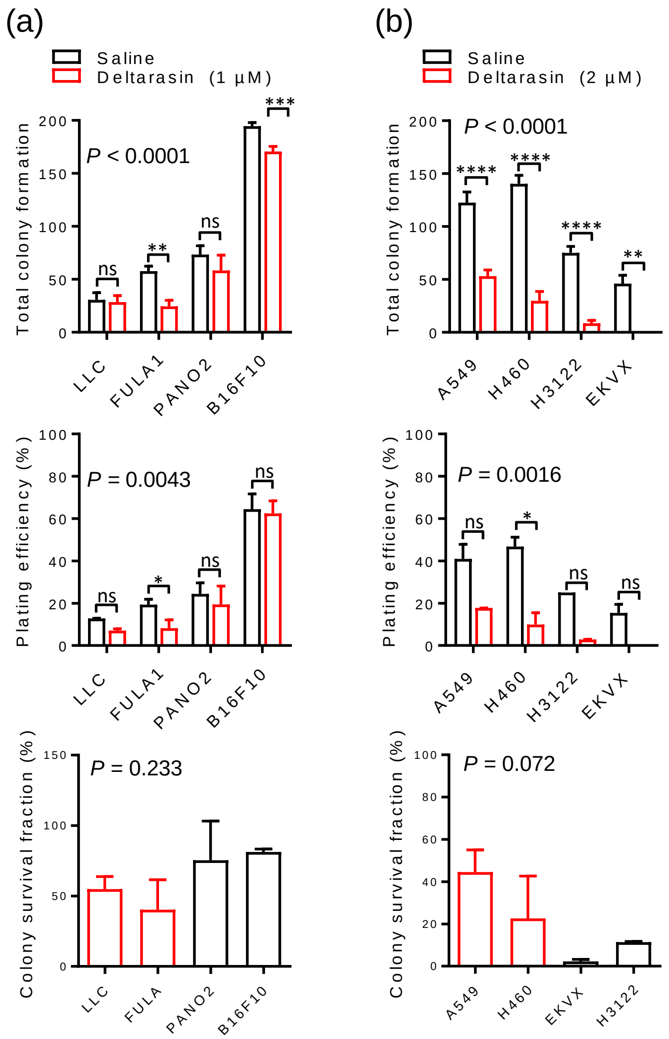

Figure S3. Response of *KRAS*-mutant tumor cells to *KRAS* inhibitors analyzed by colony formation assay.

Different mouse (**a**; *Kras*^{MUT}: LLC, FULA1; *Kras*^{WT}: B16F10, PANO2) and human (**b**; *KRAS*^{MUT}: A549, H460; *KRAS*^{WT}: EKVX, H3122) tumor cell lines were assessed for colony formation (n = 3/ data-point) after 72 h of saline or deltarasin treatment.

Data presented as mean ± SD. P, overall probability by one-way ANOVA. * and ***: $P< 0.05$ and $P< 0.001$, respectively, for the indicated comparisons by Bonferroni post-tests. Shown are total number of colonies formed (top), plating efficiency of 300 cells/well at experiment start (middle), and survival fraction of single cells given as ratio treatment/no treatment.

Supplementary Figure S4

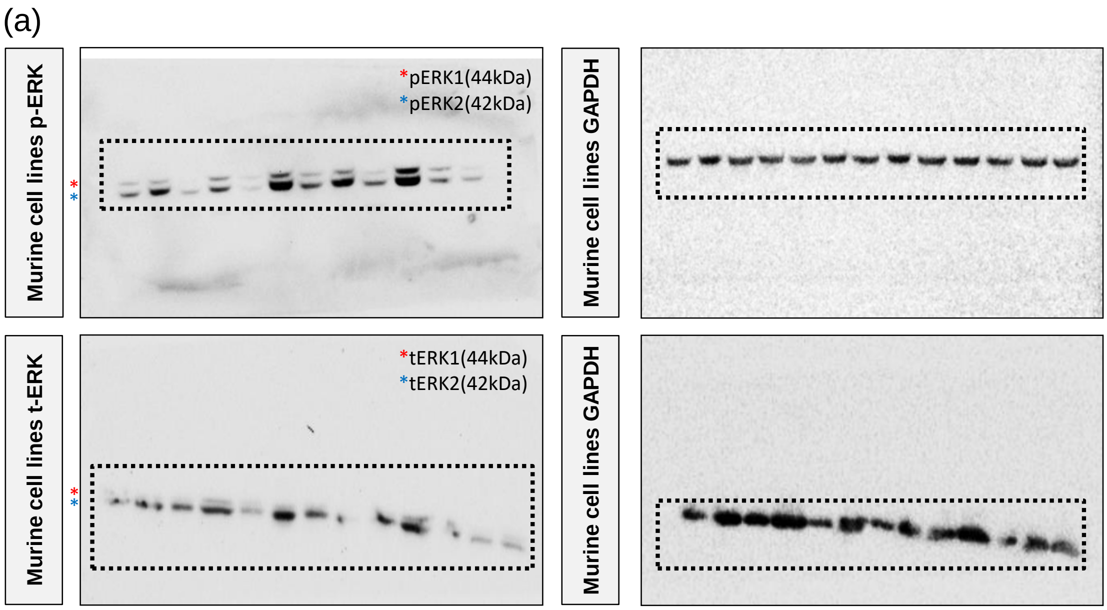

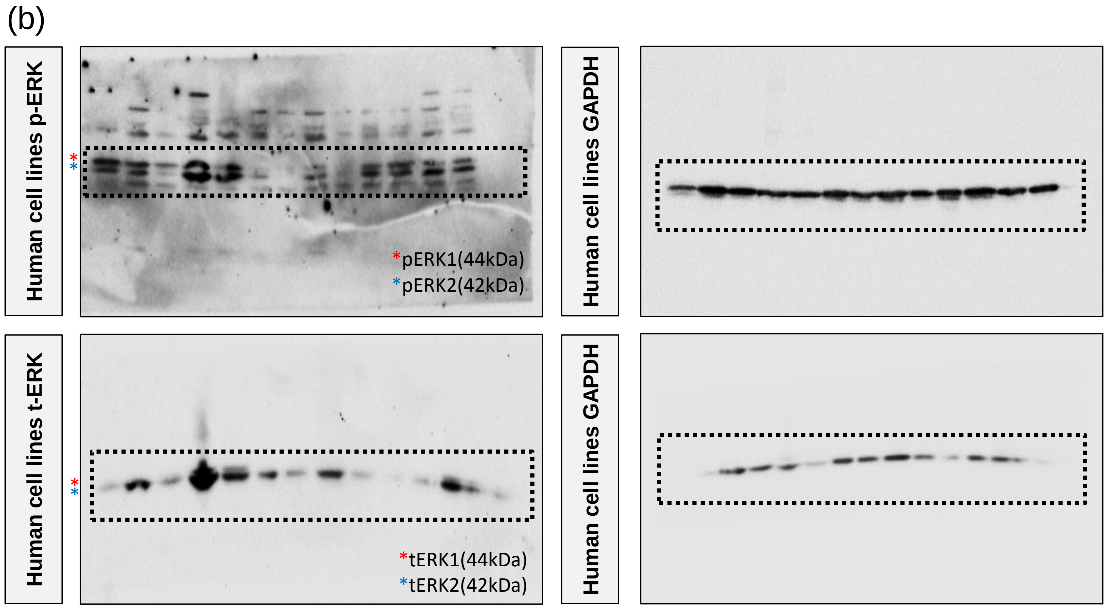

Figure S4. Uncropped blots for Figure 1h.

(a) Immunoblots of murine cell line protein extracts untreated and treated with deltarasin (72 h; IC_{60}). Left, p-ERK, t-ERK; right, GAPDH.

(b) Immunoblots of human cell line protein extracts untreated and treated with deltarasin (72 h; IC_{60}). Left, p-ERK, t-ERK; right, GAPDH.
Dashed lines represent areas of the blots shown in main Figure.

Supplementary Figure S5

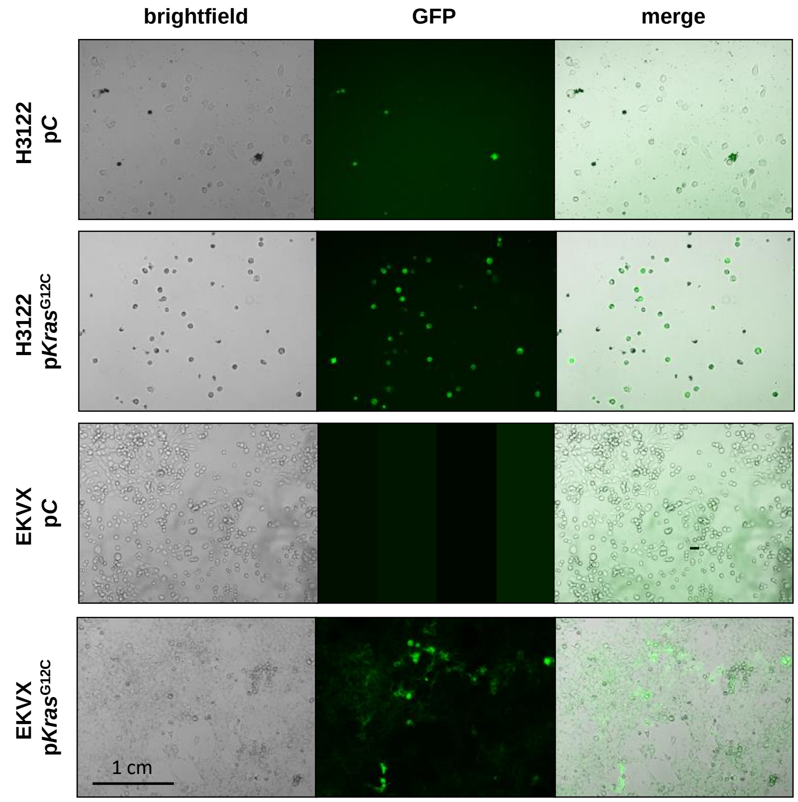

Figure S5. Validation of p*Kras*^{G12C} transduction in human cell lines H3122 and EKVX.

Thep*Kras*^{G12C}plasmid includes GFP and puromycin resistance genes. Representative microscopy images of p*C* control or p*Kras*^{G12C} transfected cell lines. Left, bright field images; middle, green fluorescent images; right, merged images. Images were taken with a confocal microscope LCI510 (Zeiss; Jena, Germany).

Supplementary Figure S6

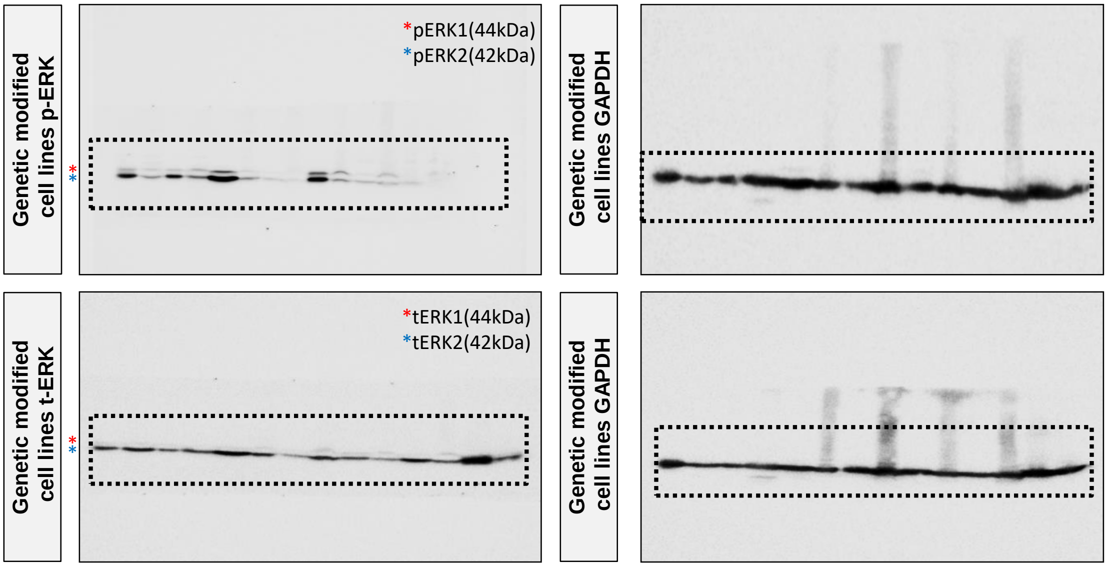

Figure S6. Uncropped blots for Figure 3d.

Immunoblots of murine and human cell line protein extracts with or without *Kras/KRAS* genetic modification. Left, p-ERK, t-ERK; right, GAPDH. Dashed lines represent areas of the blots shown in main Figure.
